# Improving recruitment to a study of telehealth management for COPD: a cluster randomised controlled ‘study within a trial’ (SWAT) of a multimedia information resource

**DOI:** 10.1186/s13063-019-3496-z

**Published:** 2019-07-24

**Authors:** Kate Jolly, Manbinder Sidhu, on behalf of the PSM COPD Group, Peter Bower, Vichithranie Madurasinghe, MRC START Group, Sandra Eldridge, Jonathan Graffy, Adwoa Parker, Peter Knapp, David Torgerson, Shaun Treweek

**Affiliations:** 1 Institute of Applied Health Research, University of Birmingham, Birmingham, B15 2TT UK; 2 Health Services Management Centre, School of Social Policy, University of Birmingham, Birmingham, B15 2RT UK; 3 NIHR School for Primary Care Research, Centre for Primary Care and Health Services Research, University of Manchester, Manchester, M13 9PL UK; 4 Centre for Primary Care and Public Health, Blizard Institute Yvonne Carter Building, 58 Turner Street, London, E1 2AB UK

**Keywords:** Recruitment, Patient information, Research methodology, Randomised controlled trial

## Abstract

**Background:**

Good quality information is critical for valid informed consent to trials, but current paper-based consent procedures are potentially unwieldy and can be difficult to comprehend, which may deter people from participating. Multimedia resources may be able to provide more accessible and user-friendly information.

We aimed to test whether offering access to a multimedia information resource alongside standard, printed patient information impacted on recruitment rates by conducting a pragmatic ‘study within a trial’ (SWAT) embedding a trial of a multimedia resource within an existing trial.

**Methods:**

The PSM COPD study involved people with mild symptoms of chronic obstructive pulmonary disease (COPD) recruited from primary care being randomised to a nurse-delivered telephone health coaching intervention, or usual primary care.

For the SWAT of recruitment procedures, practices recruiting participants were cluster randomised to use either the standard printed patient information materials or standard printed patient information materials with access to a multimedia information resource.

The multimedia resource was developed by patient and public involvement (PPI) contributors and researchers, and included *study-specific information* (e.g. study purpose, risks), and *generic information* about trials (e.g. confidentiality, randomisation). We developed a list of components and used animations as well as video clips of patients discussing their experiences of participation, matched to these components.

The primary outcome was the proportion of participants randomised.

**Results:**

Nine point six percent of those receiving standard printed patient information materials and access to the multimedia information resource were recruited, compared to 10.8% in those receiving standard printed materials alone (odds ratio (OR) = 0.844, 95% confidence interval (CI) 0.58 to 1.22).

We also found no effects on the proportion of people responding to the invitation (OR = 1.02, 95% CI 0.79 to 1.33) or retention in the trial at 6 (ORs 0.84, 95% CI 0.57 to 1.22) and 12 months after randomisation (ORs 0.80, 95% CI 0.54 to 1.18), respectively.

**Conclusions:**

The study suggests no benefits of access to a multimedia information resource alongside patient information materials on recruitment. This may reflect the limited engagement of patients with the multimedia resource. Further uses of multimedia resources will need to explore how content can be explicitly matched to user needs and preferences and methods to encourage engagement to see if effects can be enhanced. More SWATs of multimedia into ongoing trials will provide a more precise estimate of effect, and explore further how effects vary by trial context and recruitment process, intervention, and patient population.

**Trial registration:**

Current controlled trials ISRCTN 06710391. Registered on 21 November 2013.

**SWAT registration:**

SWAT 23: Systematic Techniques for Assisting Recruitment to Trials (MRC START). Registered on 11 January 2012.

**Electronic supplementary material:**

The online version of this article (10.1186/s13063-019-3496-z) contains supplementary material, which is available to authorized users.

## Background

Participant recruitment is essential for the delivery of trials, but many trials fail to recruit to time and target [1, 2]. Despite these problems and their effects on trial validity and costs, there is little rigorous quantitative research to support recruitment efforts. Embedding trials of different recruitment methods in trials (so called ‘studies within a trial’ or SWATs) is an effective way of testing methods [3] and is increasingly supported by funders [4], but a recent Cochrane review identified only 68 studies of this type [5].

### Enhancement to patient information – the role of multimedia

A conventional method of recruitment is providing information to potential participants to help them make an informed decision. Conventional methods are largely paper-based. There are concerns about the quality and comprehensibility of standard paper presentations [6, 7], which in part reflect their lack of flexibility – as there are far fewer options to present information in ways that are engaging and informative, or matched to the needs and preferences of users.

Multimedia interventions may offer a useful way forward, as they provide a useful platform for health communication, including allowance for self-directed and tailored learning [8, 9], greater user choice and potential for personalisation, and may better meet the needs of an audience increasingly used to obtaining information digitally.

Reviews of the impact of multimedia interventions on research participation have explored a variety of outcomes, including knowledge and understanding, recall, willingness to participate, perceptions of the value of research, as well as decision-making outcomes. Only a small number of studies explored the effects of multimedia materials in the Cochrane review on improving recruitment to trials [5], and the overall conclusion was of uncertainty concerning the effects. Given the limited evidence base and the ubiquity of multimedia, further research is clearly warranted.

### Testing the effects of multimedia interventions

The ‘Systematic Techniques for Assisting Recruitment to Trials’ (START) programme seeks to increase the evidence base in this area by developing a platform to encourage the rapid and rigorous testing of recruitment interventions by conducting SWATs in host trials [10].

As part of the START programme, we recruited trials funded by the UK National Institute of Health Research Health Technology Assessment Programme or registered with the Primary Care Research Network portfolio. Host trials were offered access to one of two interventions: optimised participant information materials [11] or multimedia information presented via the Internet, both intended to improve communication of trial information to potential participants.

### Aims

This study aimed to determine whether access to a multimedia information resource alongside standard printed patient information improved recruitment, compared to standard printed patient information alone.

## Methods

The study was reported in line with published guidance [12].

### Description of the PSM host trial

The host trial was called ‘PSM COPD’, and was a pragmatic multicentre trial of telephone health coaching to support self-management compared with usual care for people with COPD with mild dyspnoea. The protocol and main results paper for the host trial have both been published [13, 14]. Patients were recruited from 71 general practices around Birmingham, Oxford, Manchester, and Stoke-on-Trent. Patients had to be aged 18+ years and to meet the following eligiblity criteria: (1) on the practice COPD register, (2) experience mild dyspnoea (Medical Research Council (MRC) grades 1 or 2), (3) had an FEV_1_/FVC < 0.7 after post-bronchodilator spirometry.

Eligible patients were sent a letter from their general practitioner, with a slip for return to the research team. Interested patients were telephoned by the research team for further assessment and informed consent.

### Description of the SWAT

To ease the logistics of the trial, practices (cluster level) in the host trial were randomised using stratified (by area – Birmingham, Oxford, Manchester, and Stoke-on-Trent) block randomisation (ratio 1:1, with three varying block sizes selected randomly by the computer) to access to the multimedia information resource or only the printed patient information sheet (i.e. all patients from a particular practice were sent the same invitation letter). All patients identified as potentially eligible for the PSM host trial were eligible for the SWAT; there were no additional eligibility criteria for the SWAT. To ensure allocation concealment, the allocation sequence was generated centrally by VM (who had no other involvement in the running of the host trial) using the ‘ralloc’ command in Stata. Although informed consent was gained from patients in the host trial, patients were not aware that they were being randomised within the SWAT and no formal consent was taken. As noted above, we provided a link to the multimedia resource, but the decision to access the resource was entirely that of the patient. No changes to methods were made after commencement.

Due to the logistics of the study, only 58/71 (82%) of the practices taking part in the host trial undertook the SWAT. The host trial ran from 2013 to 2016. There was no pre-study sample size calculation for the SWAT.

### Development of the multimedia SWAT intervention

Intervention content was informed by four elements:

1. Core components for the multimedia information resource were generated by team members
2. A review of factors identified by patients as determinants of decisions about trial participation was undertaken
3. The multimedia information resource had input from members of a patient and public involvement (PPI) forum
4. The multimedia information resource had input from qualitative experts on patient health experiences (http://www.healthtalk.org/)

Multimedia interventions offer a platform for learning which can include *study-specific information* (e.g. study purpose, risks), and *generic information* (e.g. confidentiality). Patient and public involvement (PPI) forum members and qualitative experts developed study-specific components involving bespoke themes such as investigator details and benefits of participation. Generic information components included information on informed consent, randomisation, and confidentiality. Existing video clips of patients discussing their experiences of participation were edited for length and carefully matched to these components. The multimedia intervention was developed by a commercial company for use on a range of platforms including desktops and smartphones. Additional file 1 shows screenshots from the multimedia intervention, showing the introduction screen, and the screens related to study-specifc and generic information.

Access to the multimedia resource was provided as part of the patient information sheet, with a URL link and QR code to assist with easy access (see Additional file 2 for the presentation of the resource to patients). However, accessing the multimedia information resource was entirely voluntary.

### Data analysis

Analyses were conducted in line with a standard statistical plan developed at Barts and the London Pragmatic Clinical Trials Unit by SE and VM (details available from the authors). Preliminary graphical and tabular examination of the data explored baseline comparability of trial arms and representativeness of the sample in terms of the overall eligible population. The primary outcome was recruitment rate, defined as the proportion of patients actually recruited to the host trial following an invitation and randomised to each group. Analysis was by intention-to-treat. The numbers responding to the trial invitation, as well as 6 and 12 months’ retention rates were secondary outcomes. Outcomes were first described separately by arm, and then compared using logistic regression to estimate the between-group odds ratio and corresponding 95% confidence interval on the basis of the intention-to-treat principle. All analyses took account of the clustering of data due to the allocation procedure by incorporating a dispersion parameter in the model and were conducted using Stata version 12.1. The stratification factor (area) was included as a fixed effect variable in the model.

Post hoc, we estimated the cost per additional participant associated with the intervention. We estimated the cost of the intervention itself based on the price paid by the research team. We estimated the potential effectiveness of the intervention from the upper limit of the 95% confidence interval. We calculated the cost per person approached, and the additional cost associated with this benefit.

### Research Ethics Committee approval

The START programme and the individual SWATs within it were approved by the National Research Ethics Service (NRES) Committee, Yorkshire and the Humber – South Yorkshire (Ref: 11/YH/0271) on 5 August 2011. As noted earlier, although informed consent was gained from patients in the host trial, we obtained ethical approval such that patients were not aware that they were being randomised within the SWAT and no consent was taken.

## Results

**Fig. 1:**
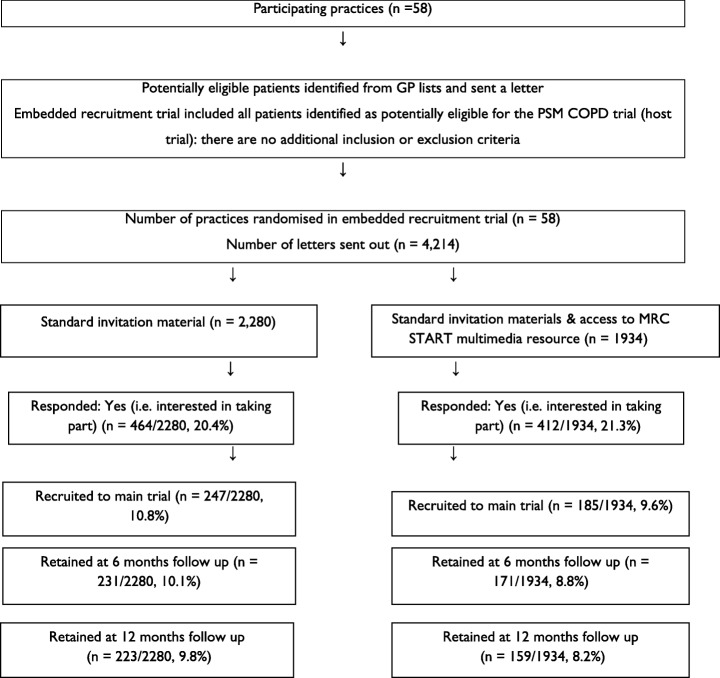
Participant flowchart

The flow of patients through the trial is shown in Fig. 1. Due to the cluster design, baseline data on patients were not available for comparison. Over 4000 patients were approached, and the rates of response to invitation, randomisation and retention over the 12 months are shown. Analyses (Table 1) showed that randomisation and retention rates were lower in the multimedia information resource group, although none of the differences reached statistical significance.

**Table 1:** Effect of interventions

Outcome	Intervention				Differences in proportions (95% CI)	^a^Odds ratio (95% CI)
	Standard invitation material		Multimedia resource			
	No.	%	No.	%		
Responded to invitation	464/2280	20.4%	412/1934	21.3%	0.0095 (−0.015 to 0.341)	1.024 (0.787 to 1.333)
Randomised to main trial	247/2280	10.8%	185/1934	9.6%	−0.013 (− 0.031 to 0.006)	0.844 (0.584 to 1.218)
Retained at 6 months’ follow-up	231/2280	10.1%	171/1934	8.8%	−0.013 (− 0.031 to 0.005)	0.836 (0.571 to 1.224)
Retained at 12 months’ follow-up	223/2280	9.8%	159/1934	8.2%	−0.016 (− 0.033 to 0.002)	0.799 (0.542 to 1.178)

*CI* confidence interval

^a^odds ratios are adjusted for stratification factor – area

We estimate that the cost of the multimedia was around £2500. We approached 4223 people (approximately £0.60 per person). Assuming the best estimate of effectiveness (a 0.6% increase), the intervention would recruit an additional six people per 1000 approached at a cost of £100 per additional patient.

## Discussion

Despite the ubiquity of multimedia and digital information, there is limited evidence so far that its benefits can be harnessed to improve patient understanding of trials, or improve randomisation and retention [5, 15]. We tested the effects of access to a multimedia information resource on recruitment and retention to a host trial evaluating a health coaching intervention for COPD. Access provided in addition to a standard patient information sheet did not improve rates of randomisation and retention. Even assuming the most favourable impacts, the cost per additional patient was around £100, which is unlikely to be cost-effective compared to simply increasing the overall numbers of patients mailed.

### Limitations

As with most SWATs, there was no formal sample size calculation, and we undertook the SWAT on the basis of the maximum number of patients and practices possible given study logistics. The cluster design would also potentially have reduced precision, although this was considered a reasonable compromise, given the potential for mistakes in allocation that may have occurred with individual randomisation across multiple sites. Although we planned to assess use of the multimedia, an error in the web-hosting software meant that we were unable to collect accurate data on use. Although this makes it impossible to assess use of the multimedia, this pragmatic trial was designed to assess the offer of access to multimedia within routine trial informed consent procedures.

Baseline data were not available for patients invited to the SWAT, as opposed to those in the trial itself. As there were no additional eligibility criteria for the SWAT, the characteristics of groups randomised in the SWAT should be similar to those in the main trial, which are detailed in the main publication [14]. In summary, the population was mostly male, with a mean age of 70 years, with limited educational qualifications and with most retired from work. We were unable to assess whether those recruited by multimedia differed in characteristics from those who were recruited using conventional methods [16]. Such analyses are not routinely done in published SWATs [11, 17]. The likely modest impact of many recruitment interventions means that the effects on the types of patients recruited is likely to be minimal, although this may be important to report in SWATs where possible.

### Interpretation of the findings in the context of the wider literature

The SWAT has been conducted in the context of a specific patient population and a specific intervention, and care must be taken in generalising the results. As part of the START programme we have embedded the same intervention in multiple host trials to better assess the effects through pooling of data, and new trials will report in due course. It is possible that the effects of multimedia information are dependent on population age and other demographic factors. As noted earlier, the population in the host trial was largely retired males aged over 70 years. The evidence suggests that Internet non-users are more likely to be women, but that non-users are also much more prevalent in those aged over 75 years [18]. The host trial population might not have been optimal for testing of this intervention. A related study is being conducted which is exploring multimedia in children and adolescents with long-term conditions. This population may be especially amenable to multimedia [19]. Future studies might also include more proximal outcomes of the multimedia intervention, such as knowledge or understanding of the trial. A recent systematic review of audio-visual information to inform potential trial participants reported small beneficial effects on patient knowledge and understanding of the trial, but no effects on trial recruitment rate, although half of included studies concerned hypothetical not real trials [20].

### Implications for recruitment practice

Although the multimedia information resource was potentially more accessible and engaging than the printed information, it would potentially take a patient more time to understand than the printed resource. If patients already find the conventional patient information sheet to be complex and take a long time to read, they may not find additional information useful, even if the presentation is more engaging.

As noted, the trial procedures meant that we were only able to provide patients with a link to the multimedia resource and not more actively encourage its use. Due to an error in coding, data on uptake or use of the multimedia resource were not available. Therefore, it is not clear whether the resource was not accessed at all, or whether it was accessed and ineffective, or whether it had variable effects (increasing participation in some patients, and reducing it in others).

Another important consideration is the context of the study and the methods of recruitment. Mailing letters from primary care is a common and reliable strategy, but means that getting multimedia into the consent process is difficult. Studies where the initial contact is face to face or by telephone with a researcher may be much more fruitful contexts for testing the results of multimedia.

It is also possible that access to accurate information leads to positive benefits on patient understanding [20], but that this does not translate to improved recruitment (or even reduces it). In-depth qualitative work alongside the SWAT would have been useful to explore whether it matched the needs of users, as well as use and interpretation of the resource, but this was beyond the resources of the project.

## Conclusions

Access to a multimedia information resource had no important effect on recruitment or retention to a host trial. Further SWATs of this technology, exploring effects in different population, are required, alongside innovation in the ways in which patients can access and use such resources.

## Additional files

Additional file 1: Example screens from the multimedia resource. (DOCX 1068 kb)

Additional file 2: Presentation of the resource to patients. (DOCX 93 kb)

## Data Availability

The datasets used and/or analysed during the current study are available from the corresponding author on reasonable request.

## Abbreviations

MRC: Medical Research Council
START: Systematic Techniques for Assisting Recruitment to Trials
