# Supplementary material for: Improving recruitment to a study of telehealth management for COPD: a cluster randomised controlled ‘study within a trial’ (SWAT) of a multimedia information resource
Source: Trials. 2019 Jul 24;20:453. doi: 10.1186/s13063-019-3496-z (PMC6657092; doi:10.1186/s13063-019-3496-z)
Supplement: Supplementary file 1 — Example screens from the multimedia resource. (DOCX 1068 kb) [file 13063_2019_3496_MOESM1_ESM.docx]

Front screen


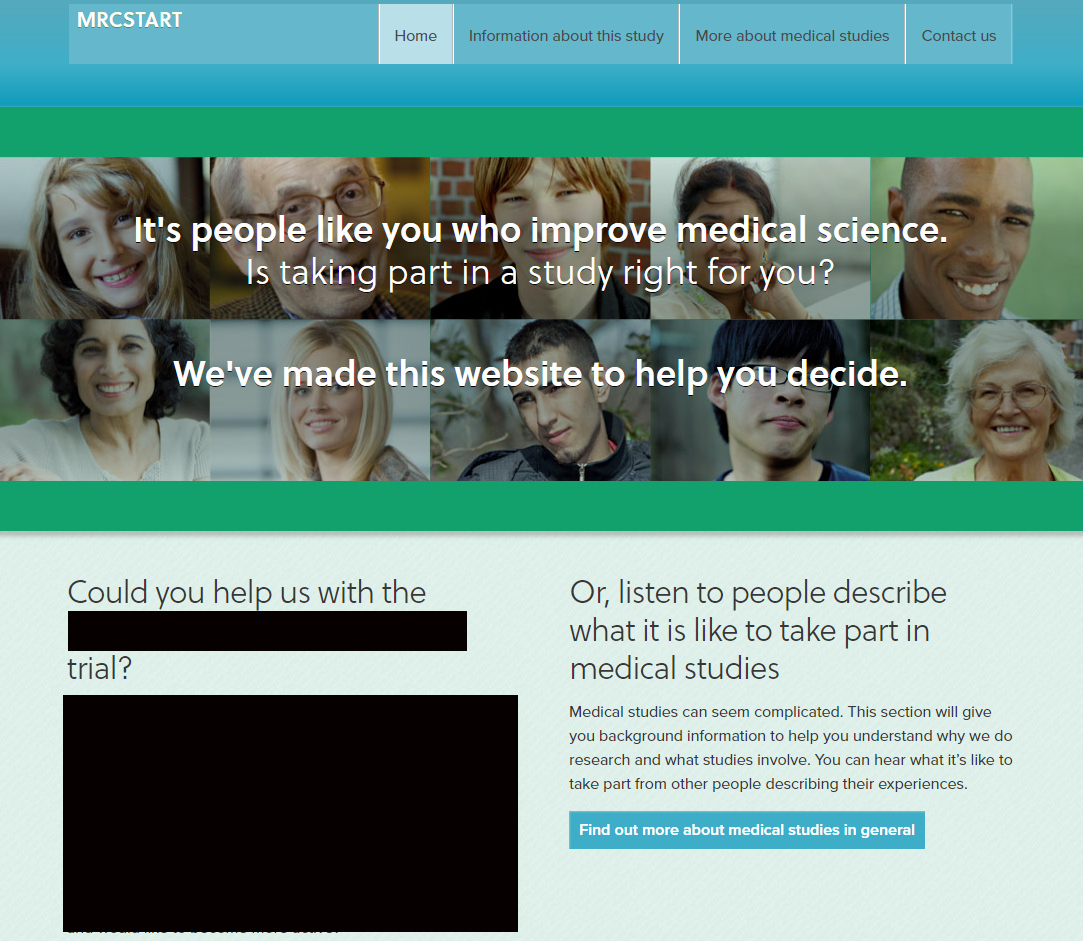


Note: The redacted areas would include details of the specific study

Study information screen


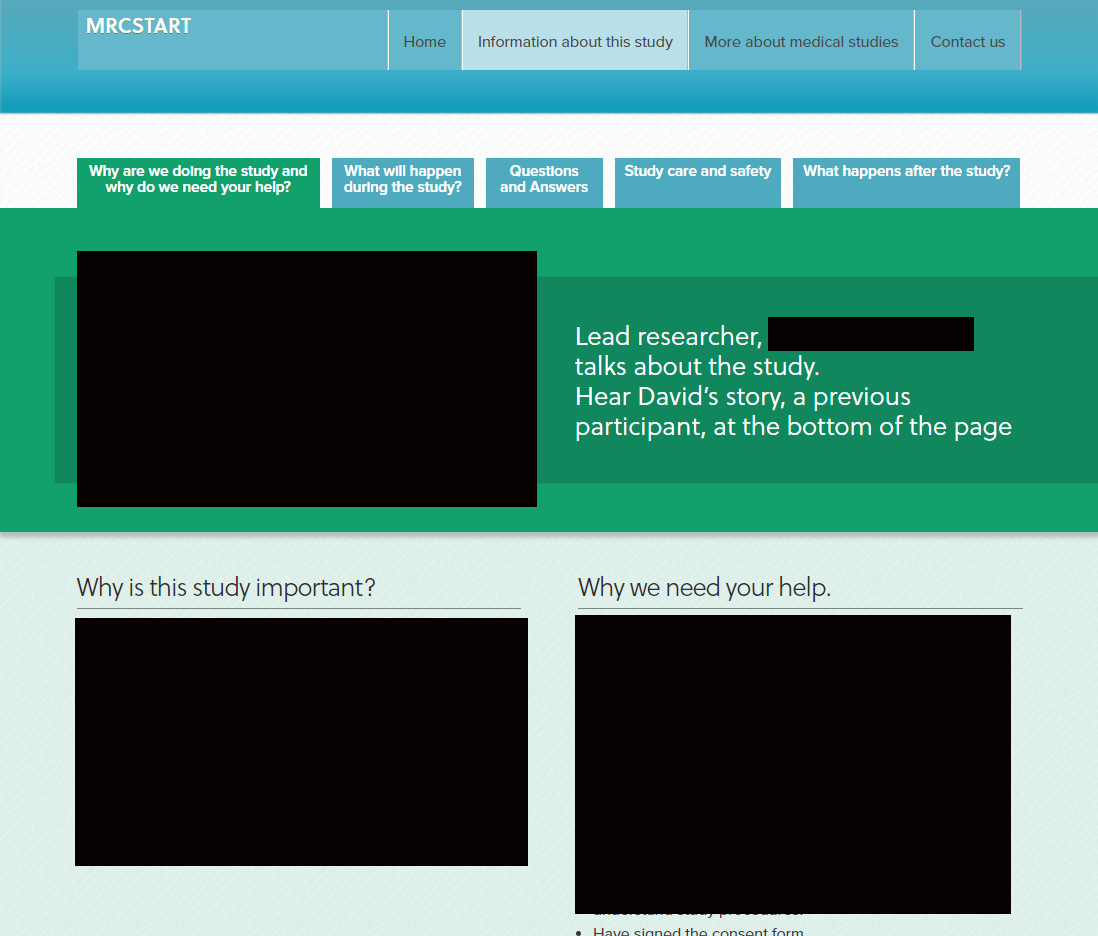


Note: The redacted areas would include details of the specific study, including a video clip from the principal investigator

Information about medical studies


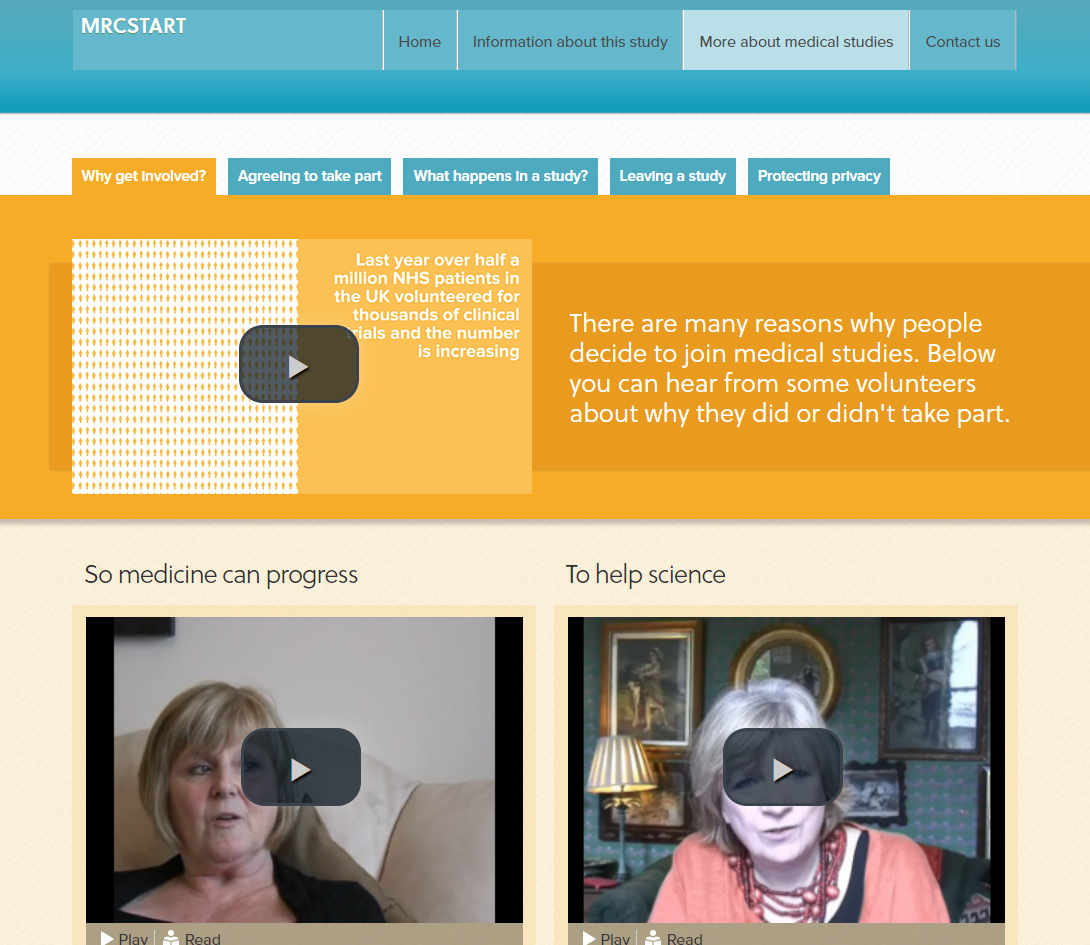


Note: The videos are from <http://www.healthtalk.org/> and are used with permission
